# Circular Economy and Sustainable Recovery of Taiwanese Tilapia (*Oreochromis mossambicus*) Byproduct—The Large-Scale Production of Umami-Rich Seasoning Material Application

**DOI:** 10.3390/foods12091921

**Published:** 2023-05-08

**Authors:** Chia-Hua Lin, Ying-Tang Huang, Jhih-Ying Ciou, Chiu-Min Cheng, Guan-Ting Wang, Chun-Mei You, Ping-Hsiu Huang, Chih-Yao Hou

**Affiliations:** 1 Ph.D. Program of Aquatic Science and Technology, College of Hydrosphere Science, National Kaohsiung University of Science and Technology, Kaohsiung 811, Taiwan; chiahua817@gmail.com; 2 Department of Marine Biotechnology, National Kaohsiung University of Science and Technology, Kaohsiung 811, Taiwan; ythuang@nkust.edu.tw; 3 Department of Food Science, Tunghai University, Taichung City 407, Taiwan; jyciou@thu.edu.tw; 4 Department and Graduate Institute of Aquaculture, National Kaohsiung University of Science and Technology, Kaohsiung 811, Taiwan; cmcheng@nkust.edu.tw; 5 Department of Seafood Science, College of Hydrosphere, National Kaohsiung University of Science and Technology, Kaohsiung 811, Taiwan; conversekl0127@gmail.com (G.-T.W.); b83418000@gmail.com (C.-M.Y.); 6 School of Food, Jiangsu Food and Pharmaceutical Science College, No. 4, Meicheng Road, Higher Education Park, Huai′an 223003, China

**Keywords:** tilapia, umami, circular economy, byproduct, flavor, sensory evaluation

## Abstract

In this study, umami-rich seasoning powder was produced from the offcuts of Taiwanese tilapia (*Oreochromis mossambicus*) by cooking concentration and spray drying of granules while yielding an abundance of glutamic acid (0.23 mg/100 g), glycine (0.10 mg/100 g), aspartic acid (0.11 mg/100 g), lysine (0.10 mg/100 g), and 11 other aminic acids. It exhibited water content (3.81%), water activity (0.3), powder yields (68.83%), and a good water solubility index (99.89%), while the particle microstructure was a spherical powder. Additionally, it received the highest overall preference score (7.53) in the consumer-type sensory evaluation compared to commercially available seasonings. This study proves that offcuts may be part of the human diet after proper processing and can be widely used to flavor savory food. The producers involved could increase their economic returns while meeting the environmental challenges. The practical contribution could create incremental value for products to critical stakeholders at each point in the tilapia supply chain with an operational guide for transitioning from inefficient to innovative circular practices.

## 1. Introduction

The ability of aquatic products to provide high-quality protein plays a vital role in the food industry [1]. However, as the annual production rises, a similar trend for its waste also applies; meanwhile, there has been a growing concern to limit waste and recycle edible food [2]. In addition, up to 35% of global aquatic food production has been estimated to be lost or wasted annually [3,4]. The food industry is constantly exploring the concept of circular economy to transform the offcuts or waste from the production process into value-added products, which is now a challenge for the food industry and researchers alike [5,6].

In recent years, there has been interested in converting the offcuts from aquatic products processing into byproducts with circulating bio-economic value [1]. However, from 1976 to 2020, the value of the fish trade grew nominally by an average of 6.9% per year (3.9% when real terms are adjusted for inflation). Simultaneously, this reflected an increase in the proportion of trade in high-value species and products undergoing processing or other forms of value addition [3]. Aquatic offcuts have been developed into feed, organic fertilizer, fishmeal, long-chain omega-3 fatty acids oil (omega-3 PUFA), protein hydrolysates, collagen, chitin, chitosan, and hydroxyapatite, etc., for food, cosmetic, and pharmaceutical materials for byproduct production [1,7,8]. These resource recycling conversions also allow for sustainable social and economic turnovers. In addition, it implies a practical model for protecting human and animal health from the environment [5,9]. Food production involves various activities ranging from formulation to safer food additives, coloring agents, new packaging systems, and nutritional foods [10,11]. Achieving sustainability in food production and trade is a contemporary challenge in the food industry [10]. A holistic approach is required to provide food security and nutrition to all generations. The chemical dependence of flavoring in food production is increasingly being challenged by these essential elements that contribute to a sustainable food system. In contrast, the various steps of processing that occur in aquatic processing factories, including grading, decapitation, and trimming and removing the heads (9–12% of total weight), offal (12–18%), bones (9–15%), scales (~5%), and skin (1–3%) that make up offcuts, cannot be commercialized [3,4,12]. Regarding the seafood processing industry, it may be possible to generate up to 90% of the byproducts from the whole organism. Unfortunately, the raw materials are susceptible to microbial growth, chemical changes, autolysis by endogenous enzymes, cross-contamination, and rapid degradation unless adequately handled or stored, and large quantities require proper management to avoid severe environmental impacts and food hygiene and safety risks [3,12]. However, the offcuts are also rich in amino acids, omega-3 PUFA, vitamins A and D and B12, carotenoids, and minerals (iron, zinc, calcium, phosphorus, and selenium) [3,4,9]. Therefore, proper handling, processing, preservation, packaging, and storage measures are essential to extend shelf life and subsequent processing. Applying suitable food processing technologies may allow offcuts to be recycled into highly nutritious products at low costs, such as fish sausages, pies, cakes, snacks, soups, sauces, and other consumer products. Thus, excellent opportunities exist to increase the nutritional impact of aquatic food resources and reduce losses and waste. Concurrently, such efforts may improve global aquatic food sustainability and green economy.

Moreover, Do et al. [13] reported that some published works have failed to provide empirical evidence of any successful practical application or convey the underlying ideological subtleties of the circular economy, such as the cascading use, where previously developed commodities and related surplus resources might be re-utilized. Kruijssen et al. [12] also reported that most studies were experimental, thus giving the impression that they are of limited use for policy recommendations. However, the impact of each production stage, from laboratory scale to scale-up, has a very different effect than real-life storage or preparation, at least in terms of quantity handled, capacity, and environmental cleanliness of the gaps. Therefore, it is necessary to avoid the impractical use of the “one-size-fits-all” approach used in previous studies to distinguish bycatch [12].

FAO [3] also proposes developing new ingredients or various forms of new products based on fish byproducts to effectively add value to the product, avoid economic losses, and be environmentally friendly while offering consumers nutritious, low-cost, and convenient products food products with a more stable shelf life. Hence, this study aimed to solve the issue of recycling scraps from the soy sauce process of tilapia fish in food companies (Graphical abstract). Specifically, the offcuts were extracted with water, heated, and concentrated for enhancing the number of solids, while spray drying was used to explore the optimum conditions for producing umami-rich seasoning byproducts with sensory evaluation. The goals were to effectively reduce procurement costs, maximize the effectiveness of raw materials, and create a sustainable circular economy.

## 2. Materials and Methods

### 2.1. Materials

Taiwanese tilapia (*Oreochromis mossambicus*)-flavored soy sauce and offcuts were purchased from the Pin-An Industrial Co. (Yuanlin County, Taiwan). All fish offcuts were packaged in polystyrene boxes while maintaining the temperature (~4 °C) with ice cubes and were delivered to the laboratory within 2 h. All offcuts were used for this study without any food spoilage. Unless otherwise specified, all chemicals were purchased directly from MilliporeSigma^®^ or Sigma-Aldrich^®^ (Merck KGaA, Darmstadt, Germany). Food-grade ingredients (maltodextrin and salt) were purchased from local supermarkets (Kaohsiung, Taiwan).

### 2.2. Pre-Processing of Samples

Processed offcuts were collected from a fish-flavored soy sauce factory. Each batch of 50 kg was loaded into a stainless-steel pot (200 L) with 50 L of reverse osmosis (RO) water and cooked for 48 h (maintained at 75 °C) to enhance the number of soluble solids by up to 70% of weight reduction. After filtration, the clarified tilapia soup stock (58%), fish-flavored soy sauce (23%, commercialized from a previous study), and salt (19%) were mixed, followed by spray drying with a spray granulator (Model YS-FDG-2, Yunlin, Taiwan). To select the carrier for the spray-dried umami-rich seasoning powder, maltodextrin, lactose, and indigestible dextrin were tested in a pre-test. The goal was to obtain the powder’s best color and microstructure performance with the carrier’s lowest cost.

### 2.3. Experimental Design of Response Surface Methodology (RSM) for the Umami-Rich Seasoning

This study used Design-Expert software (ver. 10, Statease Inc., Minneapolis, MN, USA) to generate the experimental design and analysis with response surface methodology (RSM) for statistical and regression analyses. Three levels of response surface analysis, based on Box and Behnken [14], were used to determine the production process parameters. The independent variables were as follows: A (amount of maltodextrin (300–500 g)), B (flow rate of the feed pump (2, 4, and 6 rpm)), and C (inlet temperature (60, 70, and 80 °C)) (Table A1). Fifteen runs were randomized to minimize the effects of unexpected variability on the final product. The obtained finished products were used for further analysis.

### 2.4. Basic Composition Analysis

The protein (%), fat (%), moisture (%), and ash (%) contents of the samples were determined using the method described by the AOAC [15] standard method performance requirements (SMPR^®^); the specific methods were 2016.013, 991.36, 950.46, and 942.05, respectively.

### 2.5. Physicochemical Analysis

Sugar content was measured using a handheld refractometer (N-1E ATCO Co., Tokyo, Japan) and expressed as °Brix. Salinity (g/100 g) was measured using a handheld salinity meter (Sinar Salt Meter, Merbabu Co., Osaka, Japan). For pH analysis, the sample was diluted, and 50 mL was measured at room temperature with a pH meter (SP 2500, Suntex Instruments, New Taipei City, Taiwan). Water activity (AW) was measured in a sealed, constant-temperature moisture activity meter (MX-50; A&D Store, Inc., Wood Dale, IL, USA) to measure the water diffusion equilibrium in the sample. All powder appearances were recorded under normal light using a 24.1 megapixel SLR camera (EOS 850D; Canon Inc., Tokyo, Japan). All of the above measurements were conducted following the manufacturer’s instructions. The chromaticity (including L*, a*, b*, and ΔE) measurement method was described by Hou et al. [16], with minor modifications for detection in powder samples using a colorimeter (Nippon Denshoku, SA2000, Tokyo, Japan).

### 2.6. Free Amino Acids

The free amino acid content was determined according to Rodrigues et al. [17] and Wang et al. [6], with minor modifications. Briefly, the tilapia soup stock sample (15 g) with 15 mL of pre-cooled 7% trichloroacetic acid (TCA) was homogenized for 2 min, then centrifuged at 4000× *g* for 20 min (4 °C), and the supernatant was filtered to prepare for use. The precipitate was extracted by repeating the above operation one time. Finally, the above-filtered supernatants were mixed and quantitated to 50 mL with 7% TCA. Additionally, 5 g of the powder sample was taken twice as described above and then eventually set to 100 mL. Next, 40 mL of the sample was placed in a separatory funnel and shaken with equal amounts of ether in a hood before removing the TCA and lipids, and the operation was repeated five times. The aqueous layer was concentrated by rotary vacuum until it was dried, dissolved by adding a small amount of distilled water, and quantified to 25 mL. Finally, the TCA extract (1 mL) was diluted with 0.02 N HCl, filtered through a 0.22 µm filter membrane, and analyzed using an amino acid analyzer (L-8900, Hitachi Co., Tokyo, Japan).

### 2.7. Determination of Water Solubility Index (WSI)

The WSI was performed according to a modified method described by Huang et al. [18] and Xu et al. [19], respectively. A powder sample (0.5 g) was placed in a 10 mL centrifuge tube, and 6 mL of distilled water was added, followed by vortexing. The samples were incubated in a constant-temperature water bath (30 °C) for 30 min with shaking (120 rpm), after which they were centrifuged at 3000× *g* for 20 min (4 °C). The supernatant was then transferred to a weighed beaker and allowed to dry at 105 °C, after which the weight of the dissolved material was measured. The following equation was performed to determine WSI:

$$\mathrm{WSI}\;\left(\%\right)=\frac{\mathrm{Soluble}\;\mathrm{solids}\;\mathrm{weight}\left(g\right)}{\mathrm{Dry}\;\mathrm{weight}\;\mathrm{of}\;\mathrm{sample}\left(g\right)}\times 100$$

### 2.8. Bulk and Tapped Densities

Bulk density typically refers to particle density as the material in the form of grains that appear in the density. However, bulk density is the volume density when the powder is poured into a container; tapped density is the density measured by shaking, tapping, and consolidating the powder; all the above densities include interparticle porosity. The method described by Amidon and Mudie [20] and Coucoulas [21] was used, with minor modifications to detect bulk and tapped densities in samples. The specific operation was as follows: bulk density was obtained by filling a dry 250 mL graduated cylinder with a known mass of powder (uncompacted, ~100 g) and then calculated with the following equation:

$$\mathrm{Bulk}\;\mathrm{density}\;\left(g/{\mathrm{cm}}^{3}\right)=\frac{\mathrm{known}\;\mathrm{powder}\;\mathrm{mass}\;\left(g\right)}{\mathrm{volume}\;\left({\mathrm{cm}}^{3}\right)}$$

The tapped density was measured with a 100 mL graduated cylinder into which 400 taps were made, followed by careful scraping of the excess powder from the top of the cylinder, and then calculated with the following equation:

$$\mathrm{Tapped}\;\mathrm{density}\;\left(g/\mathrm{mL}\right)=\frac{\mathrm{Weight}\;\mathrm{of}\;\mathrm{powder}\;\mathrm{in}\;\mathrm{the}\;\mathrm{graduated}\;\mathrm{cylinder}\;\left(g\right)\;}{100\;\left(\mathrm{mL}\right)}$$

### 2.9. Fourier-Transform Infrared Spectroscopy (FTIR)

The method described by Matwijczuk et al. [22] was slightly modified. The spectra obtained using Fourier-transform infrared spectroscopy (FTIR) (Bruker Instruments, Billerica, MA, USA) were recorded in the range of 4000–500 cm^−1^ with a resolution of 4 cm^−1^, accumulating 16 times the number of scans to determine the functional groups and structural features of umami-rich seasoning.

### 2.10. Dynamic Optical Microscope Photography Technology-Confocal

Standard operating procedures were carried out regarding the microstructure of the powder according to the operating manual provided by the manufacturer. Photographs were taken using a dynamic optical microscope photography technique (confocal) with a metallurgical microscope (Meiji MT7100; Meiji Techno Co., Ltd., San Jose, CA, USA).

### 2.11. Sensory Evaluation

Sensory evaluation was performed with modification according to the descriptions of Huang et al. [23]. The sample was obtained by mixing umami-rich seasoning powder (run 14) and a certain proportion of commercially available seaweed chips, kombu powder, and other flavoring ingredients before comparison with the sensory evaluation of commercially available homogeneous products. The present study was conducted using a nine-point scale to obtain product preferences from a consumer-based evaluation panel: extremely liked (9), enormously liked (8), liked (7), somewhat liked (6), disliked and not disliked (5), somewhat disliked (4), disliked (3), intensely disliked (2), and extremely disliked (1). The questions were designed with five evaluation items: color, flavor, umami, sweetness, saltiness, mouthfeel, and overall preference. The 50 consumer-type panels comprised employees from Pin-An Industrial Co. and students from the Department of Seafood Science, College of Hydrosphere, National Kaohsiung University of Science and Technology. The age distribution range was 20–60 years old. All samples (umami-rich seasoning powder and two commercially available products) were randomly labeled with a three-digit number for evaluation purposes. Only the project leader and associates knew the correct order. During the evaluation, the room temperature was maintained at 25 ± 2°C, and there was no noise. Each panelist was seated in a single seat with a partition while not talking or discussing with each other. Following sample tasting, the panelists rinsed their mouths with drinking water at least twice until the taste was removed, and the following sample was tasted. All completed scoring questionnaires were used for statistical analysis.

### 2.12. Statistical Analysis

All data obtained in this study were analyzed by analysis of variance (ANOVA) in IBM SPSS statistical software (ver. 20.0, IBM Co., Armonk, NY, USA). Duncan’s multiple range comparison revealed significant differences (*p* < 0.05) between the data. The resultant multinomial plots were regressed using the Surfer Access System (ver. 3.00; Golden Software Inc., Golden, CO, USA).

## 3. Results and Discussion

### 3.1. Analysis of Compositions

The raw materials used in the current study were processed offcuts collected from a fish-flavored soy sauce brewery as described above. Therefore, the liquid sample obtained by reprocessing the offcuts (described in Section 2.3) was defined as the tilapia soup stock. In addition to consumer safety, the nutritional value of food ingredients remains important for processed food. The characteristics of umami-rich seasoning were considered. Therefore, the basic compositions of tilapia soup stock, cooking liquid, and concentrated stock were analyzed (Table A2). The results showed that the first cooking liquid was obtained with a 4.8% crude protein content, which increased to 11.6% by concentration. However, the soup stock obtained from the second cooking and concentration contained 2.8% crude protein. Regarding crude fat, tilapia soup stock, cooking liquid, and concentrated fat were 0.2%, not detected, and 0.5%, respectively. The moisture content of the three samples ranged from 88–92.8%, which is associated with the properties of each liquid solution.

In contrast, the ash content was positively correlated with the above compositions, which were highest in the concentrated sample (1.1%), followed by cooking liquid (0.4%), and then lowest in soup stock (0.1%). These first-obtained components with highly nutritious fractions were used as raw materials for producing fish-flavored soy sauce. Moreover, the crude protein and fat contents of fish meat compared to the commonly known fish meat (whose primary nutrient content exists here) range from 20.0–20.4% and 1.5–1.8%, respectively, which may differ with the feeding of different diets. Nevertheless, the available variation is very small [24]. Traditional extraction methods may cause the oxidation of fish oil rich in unsaturated fatty acids [4]. The tilapia samples in this study showed lower crude fat content. Thus, this issue was negligible, and no negative feedback was received from the subsequent sensory evaluation. In the case of fat-rich fish species, risks may be considered.

### 3.2. Optimal Formulation and Conditions Using RSM for the Umami-Rich Seasoning

#### 3.2.1. Selection of Carriers

**Figure A1:**
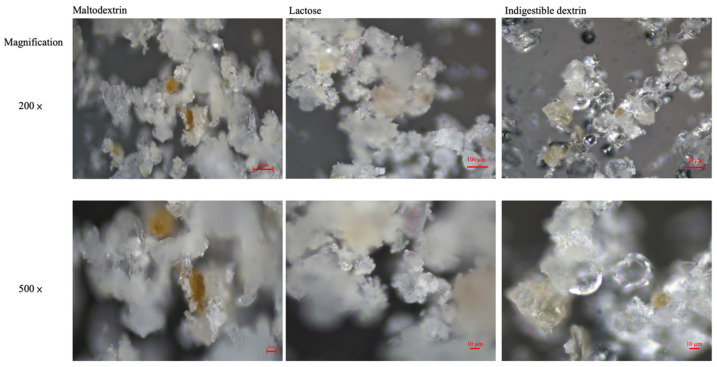
The microstructural appearance of umami-rich seasoning powder with different carriers.

In this study, the most critical factors affecting the quality indices (yield and moisture content) of umami-rich seasoning, including maltodextrin content, flow rate, and inlet temperature, were identified based on the results of preliminary trials to investigate the effects of these factors (independent variables). This study measured umami-rich seasoning powders’ chromatic and microscopic appearances with different carriers. In spray drying, the obtained single-powder particles exhibited a spherical shape (Figure A1), which is typically observed in samples with maltodextrin carriers and was in agreement with the results of Saavedra-Leos et al. [25] and Santiago-Adame et al. [26]. In the chromatic analysis, although there were significant differences (*p* < 0.05) in L*, a*, and b* among the three samples, maltodextrin was the most preferred carrier for cost-effectiveness issues (price 48.82 USD/25 kg) [27,28]. In addition, the prices of maltodextrin compared to lactose and indigestible dextrin were 60% and 35.7%, respectively (Table A3).

#### 3.2.2. RSM

Statistical regression analysis was performed using the response surface regression (RSREG) parameter in SAS based on the results of the SAS analysis. The statistical information of each variable and the independent processing variables was also obtained. The results of the response variable ANOVA based on various responses were obtained using model-fitting procedures (Table A4). The results showed that the analysis of variance in the quadratic terms B (flow rate) and C (inlet temperature) reached significant levels (*p* < 0.05), which means that doubling the amount will have a significant effect on the moisture content of the umami-rich seasoning powder. However, the ANOVA with the sequential sum of squares model (SMSS) showed an *F*-value of 5.68 and a *p*-value of 0.0352 for the regression model, indicating that the regression model was significant. Simultaneously, to confirm the validity of the examined regression model, the *F*-value for lack of fit was calculated as 1.02 with a *p*-value > 0.05, indicating no lack of a degree. Hence, this model is available. Typically, the model fit was significant (*p* < 0.05), which implies that the surface model created by quadratic polynomials is suitable for describing the variability in the quality indicators of umami-rich seasoning samples. Moreover, the coefficient of verification R^2^ reached 0.9109, which was nearly 1. In the regression calculation of the coefficients of the respective variables, the positive coefficients indicated that the moisture content of the umami-rich seasoning powder was reduced while the shelf life of the powder was improved. In the case of umami-rich seasoning powder, the quality can be explained based on RSM via an equation. Regression coefficients were obtained for each response variable, and the following three-way quadratic polynomial was obtained based on various response characteristics:

$$Y=-0.48A-0.44B+0.30C-0.08AB+0.36AC+0.47BC+0.55A^{2}+0.27B^{2}+0.09C^{2}+4.29=5.33$$

The results of the physicochemical properties obtained by RSM optimization under spray-drying conditions for the umami-rich seasoning powder (Table A5) showed that the optimum powder condition occurred at an inlet temperature of 80 °C with an input flow rate of 6 rpm. Notably, high inlet temperatures promote powder drying (reducing moisture content) while preventing adhesion in the spray-drying chamber [29]. In this case, the best-performing powder was the optimum condition for run 14, namely the umami-rich seasoning powder with the lowest moisture content (3.81%).

**Figure A2:**
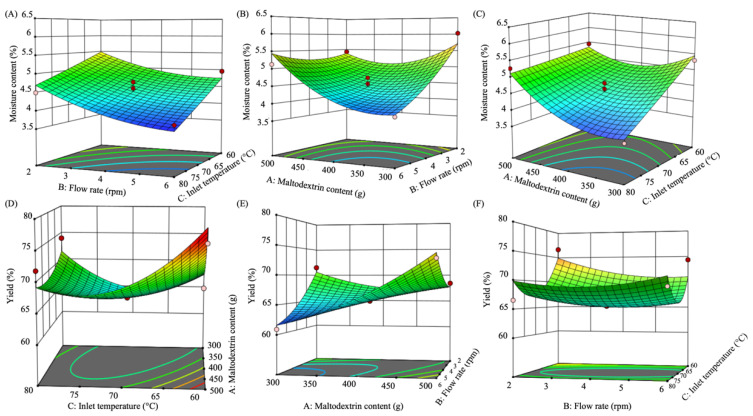
Contour plot of the regression model based on RSM polynomial for influencing factors and quality indicators (moisture content and yield). (**A**–**C**) Moisture content; (**D**–**F**) yield.

**Figure A3:**
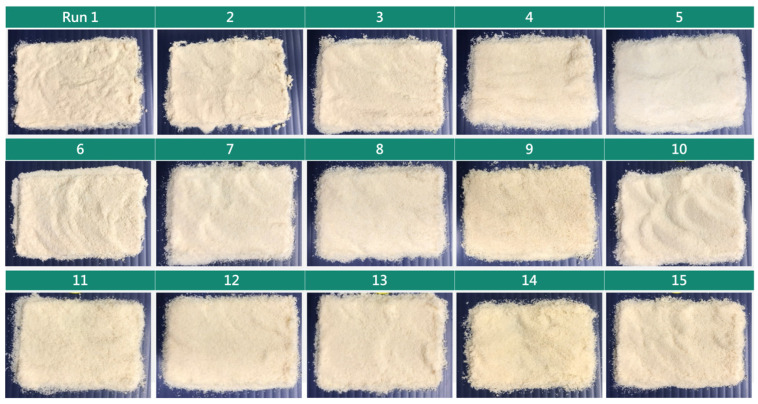
Appearance images of umami-rich seasoning powders obtained from 15 run conditions by digital camera.

The regression model of each effect factor and quality indicator (yield and moisture content) based on the polynomial obtained from RSM with isotropic plots (Figure A2) was prepared using the Surfer software package to investigate and analyze the optimal reaction conditions to investigate and analyze the physicochemical properties of umami-rich seasoning powder. In the present study, the effect of maltodextrin content on the moisture content of the umami-rich seasoning powder was influenced by the inlet temperature and flow rate of the tilapia soup stock (Figure A3A–C). Interestingly, this was attributed to the higher temperature of the inlet, which efficiently evaporated the water from the tilapia soup stock, which adhered to the carrier maltodextrin, achieving the best outcome. The inlet temperature was positively correlated with the moisture content of the umami-rich seasoning powder, which implied that it was significantly lower at higher temperatures; this result was in agreement with the findings of Rosales-Chimal, et al. [29]. There was no significant difference between the change in maltodextrin content and the increase in inlet temperature. The moisture content of maltodextrin is assumed to be comparable to that of umami-rich seasoning powders. Thus, it did not decrease during the operation. However, in the case of a thermosensitive encapsulated substance, the retention of activity correlated with the inlet temperature, feed flow, and carrier content [29]. It is worth mentioning that the maltodextrin content will be correlated with significant sensory evaluation results. Hence, this study designed the maltodextrin content as a three-factor, three-level evaluation condition for umami-rich seasoning powder.

The RSM trend showed similar results within the same level of conditions where the index setting was the yield, which also implied a significant correlation between yield, maltodextrin content, and inlet temperature (Figure A3D–F). Despite this, the maximum yield might be obtained with a low inlet temperature and high maltodextrin content. Additionally, as storage time increases, caking may occur during the shelf life, even though the moisture content remains within the requirements. Therefore, despite the encouraging possibility of obtaining higher yields with the production, as mentioned earlier, it should be noted that the feasibility of the application in the commercialization process demands additional evaluation, particularly regarding packaging materials with better containment and following rigorous storage conditions. In addition, Baltrusch et al. [30] reported that to achieve high yields of tea polyphenols, inherently high concentrations of carriers are required. Hence, there is a considerable challenge in the food industry compared to other encapsulation materials in terms of finding suitable, versatile, non-toxic, and simple operations and inexpensive carriers while meeting consumer acceptance requirements related to the final product properties (such as drying efficiency and quality) [30,31,32].

### 3.3. Physicochemical Characterization of Umami-Rich Seasoning Powder

**Figure 1:**
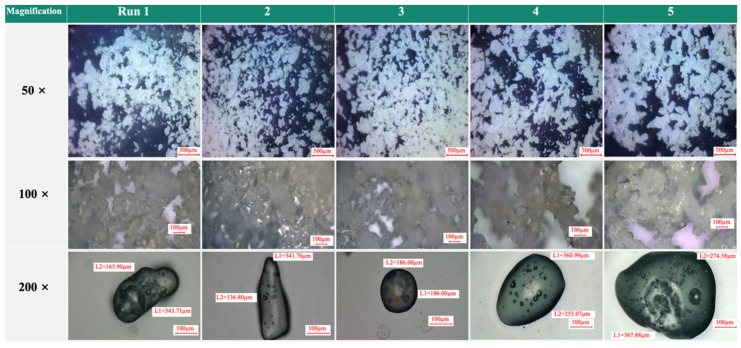

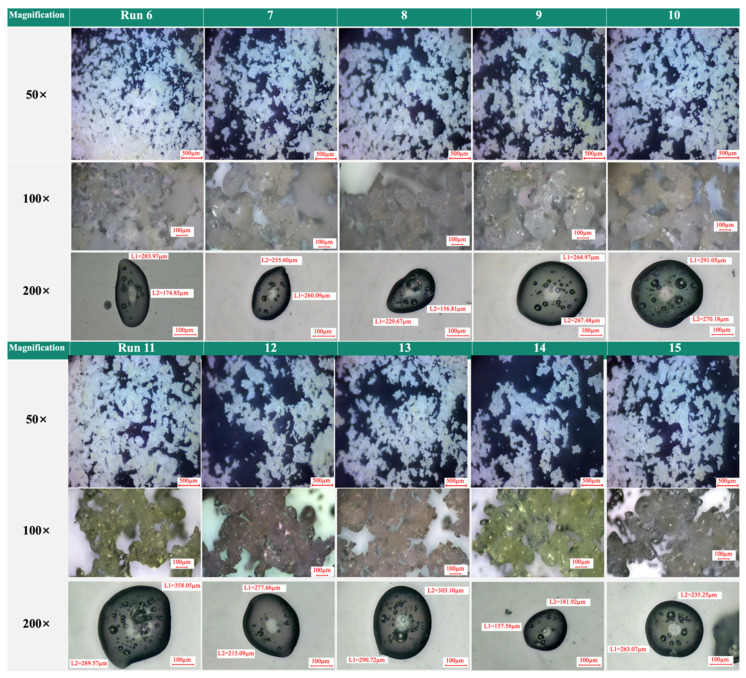
Microstructure photographs of umami-rich seasoning powders by a dynamic optical microscope (confocal with 50, 100, and 200 magnification observation, respectively).

**Figure 2:**
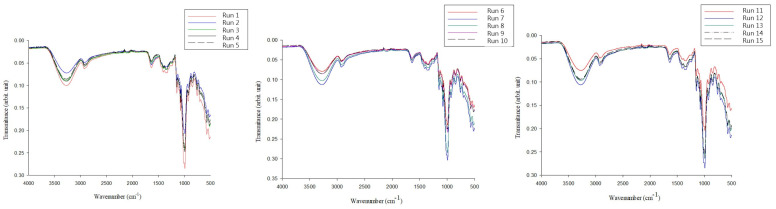
FTIR spectra of the umami-rich seasoning powders in the absorption range of 4000 to 500 cm^−1^, with 16 scans and 4 cm^−1^ resolution.

The umami-rich seasoning powder samples from the 15 groups of conditions obtained from the RSM analysis were analyzed in terms of physicochemical properties including salinity (g/100 g), saccharinity (%), pH, moisture content (%), AW, chromatic (Table 1), free amino acids (Table 2), WSI (%), bulk density tapped density (Table 3), appearance (Figure A3), microstructure (Figure 1), and FTIR (Figure 2). To avoid caking the powder in cases without other food additives, the quality index of the umami-rich seasoning powder was the moisture content. Therefore, among the 15 groups of conditions obtained from the RSM calculation, runs 6, 10, and 14 showed relatively low water contents of 3.68, 3.84, and 3.81%, respectively (Table 1), compared to other runs with significant differences (*p* < 0.05). This also implies that the operating conditions used in the present study were efficient for evaporative water drying, which was consistent with the results of Ferreira et al. [33]. In addition, the moisture content of foods under 6% have high stability and shelf life [34]. Interestingly, the AW (0.28–0.40) for all conditions was below the microbial growth level (<0.6) [33]. In terms of salinity, saccharinity, and pH, there were significant differences among each run (*p* < 0.05), though these were not considered quality indicators. However, maltodextrin is a hygroscopic material that results in a lower moisture content of the powders and with higher hygroscopicity [31,33]. The chromatic analysis (L*, a*, b*, and ΔE) of the run powders were statistically significantly different (*p* < 0.05); however, the variation was difficult to detect through visual observation (Figure A3).

Regarding the determination of free amino acids, there were no significant differences between the umami-rich seasoning powders in all the runs. However, glutamic acid (0.12–0.29 mg/100 g), glycine (0.06–0.12 mg/100 g), aspartic acid (0.06–0.14 mg/100 g), and lysine (0.06–0.11 mg/100 g) were found in higher contents than other amino acids in this study (Table 2), thus providing a richer umami flavor. These results were similar to those of the umami amino acids identified in porcine bone soup [35]. Yin et al. [36] reported that free amino acids in tilapia fillets contain five sweet amino acids (Gly, Ala, Arg, Thr, and Lys), with glycine being the most abundant. However, the amino acid contents in this study were lower than those mentioned above, which could be attributed to the fact that the samples in this study were sourced from offcuts from the fish-flavored soy sauce process.

In addition, umami-rich seasoning powder contains several amino acids, which can be used as natural seasoning agents to replace commercially available food additives such as high-flavor monosodium glutamate (MSG; L-glutamate) and seasoned chicken/mushroom essence, whose sodium content has been considered to be excessive [37]. However, each culinary culture has a unique flavor combination of seasonings and ingredients, which is why local consumer preferences are referred to as flavor principles [38,39,40]. It is worth mentioning that regardless of which culinary culture is inseparable from the umami flavor, including free amino acids (aspartic acid), 5′-nucleotides, short peptides, organic acids (such as lactic and succinic acid), and some Maillard reaction derivatives, which are widely distributed in food to enhance the overall flavor of food, for example, adjusting sweetness, enhancing salty taste, suppressing acidity, and bitterness, MSG is a classic seasoning type [37,39,41,42,43,44]. All tastes are related to each other, especially umami’s enhancement of salty taste [45], which has no adverse effects on the human body in appropriate amounts, unlike MSG [43,46]. It has a significantly stronger effect on saltiness enhancement than an MSG-free solution with 0.3% NaCl [42]. Thus, a suitable amount of umami seasoning is available to replace salt while preserving consumer acceptance of food [47]. Despite the encouraging potential, it has also been reported that excessive intake of MSG combined with a high-lipid diet may lead to systemic damage through metabolic modulation of different signaling pathways, such as dyslipidemia due to the leptin/adiponectin ratio and inflammation due to oxygen species due to altered redox homeostasis, with severe cases of apoptosis [48]. K.-C. Hsu, E.C.Y. Li-Chan, and C.-L. Jao [49] showed that small-molecule amino acid sequences from tuna hydrolysis had an inhibitory effect on the proliferation of MCF-7 cells. The bitter taste of protein hydrolysates (due to alkaline protein hydrolysis enzymes releasing specific peptides) has previously limited their application but has been partially solved by substances such as glutamate, polyphosphate, activated carbon, gelatin, or glycine [50,51,52].

In terms of powder properties, the WSI of the umami-rich seasoning powder obtained for each run condition ranged from 98.46–99.89% (Table 3), with significant differences between the runs (*p* < 0.05). In addition, the umami-rich seasoning based on maltodextrin showed a relatively better performance than the commercially available seasoning powder (bonito soup stock) (WSI 89.7). In addition, these values were significantly different from those of all the runs in this study (*p* < 0.05). Other properties of the powders, such as bulk and tapped densities, were significantly different (*p* < 0.05) as measured for the umami-rich seasoning powders obtained for each run condition, ranging from 0.40–0.63 (g/cm^3^) and 0.43–0.69 (g/mL), respectively (Table 3). Additionally, the inlet temperature rises, and the water evaporates rapidly, which forms the shell on the particle’s surface instantly, whereby the water vapor inside the particle will be released from the pores due to high pressure, which will lead to decreased bulk density [29,31,53]. Unfortunately, it was impossible to compare the differences in powder densities owing to the different drying methods used in this study and the commercially available products mentioned above.

FTIR was primarily measured by determining the difference in the spectroscopic spectra of the functional groups, which quickly and directly identified the sample’s chemical composition [22], allowing the measurement and evaluation of the effects of different processes on the sample. The images (Figure A3) of the umami-rich seasoning powders showed no significant difference in appearance between the various run conditions. These results were in agreement with those of the color analysis, albeit with significant differences in the results of the precision instrumentation, where such differences were not discerned visually. In addition, the microstructure images were taken by dynamic optical microscopy (confocal), and the microstructure profile of the umami-rich seasoning powder emerged more clearly. The results demonstrated that the microstructures of runs 1, 2, 4, 5, 6, 7, and 8 were not round, indicating that the umami-rich seasoning powder formation was not unsatisfactory (Figure 1). However, the other run conditions (3, 9, 10, 11, 12, 13, and 15) exhibited a rounded morphological structure, which failed the quality index (moisture content). However, all the samples in this study were observed to be single spherical particles agglomerated with smooth crystalline surfaces. According to Saavedra-Leos et al. [25], the inlet temperature affects the microstructure and morphology of particles. Hence, the variation in particle size and morphology may be attributed to the different processing conditions used in the spray-drying process. In contrast, run 14 had bright-yellow powder pellets and a round microstructure. Consequently, this serves as the optimum bulk production condition for umami-rich seasoning powder in this study.

The FTIR measurements of 15 run conditions of umami-rich seasoning powders showed no significant difference in the functional chemical composition of the powders obtained under each condition (Figure 2). The initial vibrational regions of all samples were observed in the 3600–3000 cm^−1^ from the FTIR spectra. These were attributed to the characteristic stretching vibrations of the -OH group of carbohydrates, water, and organic acids. The results of this study agree with those of Matwijczuk et al. [22] and L. Svečnjak, D. Bubalo, G. Baranović, and H. Novosel [54]. Łopusiewicz et al. [53] and Matwijczuk et al. [22] also reported that the vibration at 3300–3200 cm^−1^, which belongs to the irregular absorption of carboxylic acid (with a wide -OH band) while enhancing the stretching vibration of the C-H group and the wide band of vibration, is due to the strong hydrogen bonding of carboxylic acid dimer [55]. The regions of 3000–2800 cm^−1^ were characterized by stretching vibrations of the C-H groups. From 1500–1200 cm^−1^, the bands were dominated by the deformation vibrations of the O-CH and C-C-H groups in the carbohydrate structure and by the deformation vibrations belonging to the δ-OH group in the C-OH structure [33]. The range from 1200–800 cm^−1^ contains the stretching vibrations of the C-H group or (C-O) in the carbohydrate structure, while there are also C-O stretching vibrations of the C-OH group or C-C stretching vibrations in the carbohydrate structure [22,56]. Additionally, the spectral region 900–700 cm^−1^ represents anomalous regional vibrations or C-H and C-C deformations of carbohydrates, which implies relative changes in sugar bonding [57]. Thus, FTIR technology serves as a structural profiling of the chemical functional groups of samples to determine the variation [22,33].

### 3.4. Sensory Evaluation

Sensory evaluation includes the assessment of the odor, flavor, taste, texture, and appearance of food [58]. The samples for the sensory evaluation were umami-rich seasoning powder and two commercially available products, which were evaluated and analyzed statistically (Table 4). The appearance (color) score was the highest for commercially available product 2 (8.07), followed by the umami-rich seasoning powder (7.67), and then the lowest for commercially available product 1 (7.07), which was significantly different (*p* < 0.05). Flavor (odor) showed no significant difference, with umami-rich seasoning powder (7.33) being the highest, followed by commercially available products 2 (7.20), and with 1 (7.13) being the lowest. The highest score for umami was obtained for commercially available product 2 (7.80), followed by umami-rich seasoning powder (7.53) and product 1 (7.00), with no significant difference. The sweetness scores were highest for umami-rich seasoning powder (7.40), followed by commercially available product 2 (7.07), and then lowest for product 1 (6.87), with a significant difference (*p* < 0.05). Therefore, the results were attributed to the use of maltodextrin as the powder carrier in this study, which provided significant sweetness. The highest score for saltiness was umami-rich seasoning powder (7.60), followed by commercially available product 2 (7.53) and product 1 (6.73), with no significant difference. However, the source of sodium in all samples was associated with fish soup stocks. In the case of mouthfeel, commercially available product 2 (7.53) was the highest, followed by umami-rich seasoning powder (7.47), and with product 1 (6.40) being the lowest, with no significant difference. The overall acceptance ratings were highest for umami-rich seasoning powder (7.53), followed by commercially available product 2 (7.47), and then lowest for product 1 (6.73), with no significant difference. Altogether, the sensory evaluation showed that the umami-rich seasoning powder was sufficient to raise interest in consuming natural ingredients. It also confirmed the excellent commercial value of tilapia offcut soup stock and pelletizing by spray drying, which minimizes the cost of raw materials while providing additional economic benefits, especially in large-scale production. Recently, a few studies have shown that consumers prefer more “natural” foods with ingredients whose “chemical-sounding names” (as described above for umami flavor) may lead to less recognition of the naturalness of the umami-rich seasoning powder in the study [59,60]. However, compared to popular enzymatic hydrolysis processes, in this study, the raw material had more natural attributes (no bitterness masking), which provides the potential to be part of the flavoring in the human diet.

## 4. Conclusions

This study showed that the optimum conditions for large-scale production of umami-rich seasoning powder by RSM analysis were 400 g of maltodextrin, a flow rate of 6 rpm, and an 80°C initial spray drying temperature. Finally, the umami-rich seasoning powder was produced with 68.83% yield and 3.61% moisture content. The present study aimed to provide specific processes for the possible reapplication of aquaculture tilapia soy sauce residues after processing. Nevertheless, in the food industry, these products are only suitable for flavoring and promoting economic development and environmental benefits. Notably, this study perfectly resolves a specific issue: production companies’ attempts to establish customized facilitation of circular-based bioeconomic models. Although these innovative and sustainable processes have proven effective, the commercialization of byproducts still requires visibility and robust support. The evolution of a circular bio-economic production system is an innovative approach to increase employment, which is necessary for the post-COVID-19 era, through a more efficient, sustainable, and green in-house production system.

## Figures and Tables

**Table 1:** Physicochemical characterization of umami-rich seasoning powders obtained with the spray drying conditions optimized by RSM.

Run	Salinity (g/100 g)	Sugar Content (%)	pH	Moisture Content (%)	AW	L*	a*	b*	ΔE
1	73.00 ± 0.14 ^h^	97.00 ± 0.14 ^bc^	5.15 ± 0.00 ^h^	4.96 ± 0.06 ^d^	0.36 ± 0.01 ^cd^	92.70 ±0.69 ^d^	0.82 ± 0.03 ^c^	5.46 ± 0.14 ^b^	92.87 ± 0.69 ^e^
2	84.00 ± 0.00 ^abc^	100.0 ± 0.00 ^a^	4.99 ± 0.02 ^i^	5.31 ± 0.06 ^b^	0.40 ± 0.01 ^a^	92.54 ± 0.23 ^D^	1.07 ± 0.01 ^ab^	16.07 ± 0.12 ^a^	92.74 ± 0.23 ^e^
3	83.00 ± 0.14 ^bcd^	97.00 ± 0.05 ^bc^	5.40 ± 0.05 ^ef^	4.08 ± 0.11 ^f^	0.37 ± 0.01 ^c^	93.26 ± 0.39 ^cd^	1.04 ± 0.06 ^ab^	7.11 ± 0.10 ^b^	93.53 ± 0.40 ^cde^
4	83.00 ± 0.14 ^bcd^	97.00 ± 0.14 ^bc^	5.43 ± 0.03 ^cde^	5.13 ± 0.06 ^c^	0.40 ± 0.03 ^ab^	94.51 ±0.32 ^b^	0.64 ± 0.03 ^d^	10.06 ± 0.02 ^ab^	95.05 ± 0.32 ^ab^
5	86.00 ± 0.03 ^ab^	100.0 ± 0.03 ^a^	5.48 ± 0.03 ^abc^	5.33 ± 0.08 ^b^	0.36 ± 0.00 ^c^	95.72 ±0.50 ^a^	0.83 ± 0.04 ^c^	3.40 ± 0.03 ^b^	95.78 ± 0.49 ^a^
6	85.33 ± 0.03 ^abc^	99.30 ± 0.03 ^ab^	5.27 ± 0.03 ^g^	3.68 ± 0.17 ^g^	0.28 ± 0.01 ^g^	91.09 ±0.17 ^e^	1.09 ± 0.03 ^a^	4.15 ± 0.03 ^b^	91.19 ± 0.17 ^f^
7	78.00 ± 0.00 ^de^	92.00 ± 0.04 ^ef^	5.51 ± 0.04 ^ab^	4.71 ± 0.03 ^e^	0.33 ± 0.01 ^e^	90.09 ± 0.34 ^efg^	0.04 ± 0.02 ^g^	7.81 ± 0.10 ^ab^	90.43 ± 0.33 ^f^
8	80.00 ± 0.00 ^de^	94.00 ± 0.00 ^de^	5.44 ± 0.03 ^a^	5.43 ± 0.06 ^b^	0.37 ± 0.01 ^c^	94.49 ± 0.12 ^b^	0.17 ± 0.03 ^f^	3.63 ± 0.10 ^b^	94.56 ± 0.12 ^bc^
9	82.00 ± 0.00 ^cd^	96.00 ± 0.00 ^cd^	5.44 ± 0.00 ^cde^	4.49 ± 0.03 ^e^	0.33 ± 0.01 ^e^	93.20 ± 0.44 ^cd^	0.67 ± 0.14 ^d^	5.85 ± 0.21 ^b^	93.38 ± 0.43 ^de^
10	77.00 ± 0.14 ^efg^	91.00 ± 0.14 ^f^	5.42 ± 0.02 ^de^	3.84 ± 0.04 ^g^	0.30 ± 0.01 ^g^	87.93 ± 0.29 ^h^	0.97 ± 0.13 ^b^	10.62 ± 0.23 ^ab^	88.57 ± 0.31 ^g^
11	65.33 ± 0.12 ^i^	79.33 ± 0.12 ^h^	5.41 ± 0.04 ^def^	5.27 ± 0.14 ^bc^	0.32 ± 0.02 ^ef^	93.92 ± 0.92 ^bc^	0.07 ± 0.07 ^g^	3.55 ± 0.03 ^b^	93.90 ± 0.91 ^cd^
12	76.00 ± 0.23 ^fgh^	93.33 ± 0.23 ^ef^	5.45 ± 0.00 ^cde^	4.53 ± 0.21 ^e^	0.29 ± 0.00 ^g^	90.60 ± 0.29 ^efg^	−0.24 ± 0.02 ^i^	8.39 ± 0.07 ^ab^	90.99 ± 0.28 ^f^
13	86.00 ± 0.00 ^ab^	100.0 ± 0.00 ^a^	5.41 ± 0.04 ^def^	5.45 ± 0.13 ^a^	0.34 ± 0.00 ^de^	89.09 ± 0.83 ^g^	−0.83 ± 0.03 ^j^	4.75 ± 0.02 ^b^	89.22 ± 0.83 ^g^
14	74.00 ± 0.00 ^gh^	88.00 ± 0.00 ^g^	5.46 ± 0.05 ^bcd^	3.81 ± 0.09 ^g^	0.30 ± 0.02 ^fg^	90.73 ± 0.32 ^ef^	0.46 ± 0.05 ^e^	10.46 ± 0.09 ^ab^	91.34 ± 0.33 ^f^
15	87.00 ± 0.14 ^a^	100.0 ± 0.14 ^a^	5.36 ± 0.01 ^f^	6.03 ± 0.02 ^a^	0.38 ± 0.03 ^bc^	89.96 ± 0.37 ^fg^	−0.07 ± 0.04 ^h^	7.43 ± 0.20 ^b^	90.27 ± 0.38 ^f^

The values indicate the mean ± SD of triplicate determinations (*n* = 3). Different superscript lowercase letters in the same column represent significant differences between samples (*p* < 0.05).

**Table 2:** The free amino acid content of umami-rich seasoning powders obtained with the spray-drying conditions optimized by RSM.

mg/100 g	1	2	3	4	5	6	7	8	9	10	11	12	13	14	15
Aspartic acid	0.14	0.14	0.12	0.11	0.09	0.12	0.12	0.06	0.10	0.13	0.08	0.12	0.09	0.12	0.12
Threonine	0.06	0.06	0.05	0.05	0.04	0.05	0.05	0.03	0.04	0.05	0.04	0.05	0.04	0.05	0.05
Serine	0.07	0.07	0.06	0.05	0.04	0.05	0.06	0.03	0.05	0.06	0.04	0.06	0.05	0.05	0.06
Glutamic acid	0.29	0.29	0.25	0.22	0.18	0.24	0.25	0.12	0.20	0.26	0.17	0.25	0.19	0.23	0.24
Glycine	0.12	0.12	0.11	0.10	0.08	0.11	0.11	0.06	0.09	0.12	0.07	0.11	0.08	0.10	0.10
Alanine	0.11	0.11	0.10	0.09	0.07	0.10	0.10	0.05	0.08	0.10	0.06	0.10	0.07	0.09	0.09
Cystamine	-	-	-	-	-	-	-	-	-	-	-	-	-	-	-
Valine	0.07	0.07	0.06	0.05	0.04	0.06	0.06	0.03	0.05	0.06	0.04	0.06	0.04	0.06	0.06
Methionine	-	-	-	-	-	-	-	-	-	-	-	-	-	-	-
Isoleucine	0.05	0.05	0.04	0.04	0.03	0.04	0.04	0.02	0.04	0.05	0.03	0.04	0.03	0.04	0.04
Leucine	0.09	0.09	0.07	0.06	0.05	0.07	0.07	0.04	0.06	0.08	0.05	0.07	0.05	0.06	0.07
Tyrosine	0.01	0.01	0.01	0.01	0.00	0.01	0.01	0.01	0.01	0.01	0.01	0.01	0.01	0.01	0.01
Phenylalanine	0.05	0.06	0.04	0.04	0.03	0.04	0.04	0.02	0.04	0.05	0.03	0.04	0.04	0.05	0.04
Lysine	0.11	0.12	0.10	0.08	0.06	0.10	0.10	0.04	0.09	0.11	0.07	0.10	0.08	0.10	0.09
Tryptophan	-	-	-	-	-	-	-	-	-	-	-	-	-	-	-
Histamine	0.03	0.03	0.02	0.02	0.01	0.02	0.02	0.01	0.11	0.02	0.01	0.02	0.02	0.02	0.02
Arginine	0.07	0.07	0.06	0.05	0.04	0.06	0.06	0.03	0.05	0.06	0.04	0.06	0.04	0.05	0.06
Proline	0.11	0.09	0.06	0.05	0.03	0.05	0.06	0.02	0.07	0.09	0.05	0.06	0.04	0.05	0.07

**Table 3:** The results of the water solubility index (WSI), water absorption index (WAI), and bulk and tapped density of umami-rich seasoning powder.

Sample	WSI (%)	Density	
		Bulk (g/cm^3^)	Tapped (g/mL)
1	99.68 ± 0.17 ^a^	0.63 ± 0.01 ^b^	0.68 ± 0.01 ^a^
2	99.77 ± 0.16 ^a^	0.67 ± 0.02 ^a^	0.69 ± 0.02 ^a^
3	99.54 ± 0.65 ^a^	0.57 ± 0.01 ^c^	0.60 ± 0.01 ^c^
4	99.74 ± 0.20 ^a^	0.44 ± 0.02 ^de^	0.48 ± 0.01 ^de^
5	99.77 ± 0.09 ^a^	0.44 ± 0.01 ^de^	0.48 ± 0.01 ^d^
6	98.46 ± 0.84 ^b^	0.56 ± 0.02 ^c^	0.63 ± 0.01 ^b^
7	98.35 ± 0.16 ^b^	0.41 ± 0.02 ^ef^	0.44 ± 0.03 ^f^
8	99.86 ± 0.00 ^a^	0.41 ± 0.01 ^ef^	0.44 ± 0.01 ^f^
9	99.89 ± 0.06 ^a^	0.41 ± 0.01 ^ef^	0.44 ± 0.02 ^f^
10	99.78 ± 0.17 ^a^	0.40 ± 0.01 ^f^	0.43 ± 0.01 ^f^
11	99.75 ± 0.01 ^a^	0.43 ± 0.03 ^def^	0.46 ± 0.03 ^def^
12	99.80 ± 0.03 ^a^	0.45 ± 0.02 ^d^	0.49 ± 0.01 ^d^
13	99.87 ± 0.04 ^a^	0.41 ± 0.00 ^ef^	0.48 ± 0.01 ^de^
14	99.89 ± 0.03 ^a^	0.42 ± 0.01 ^def^	0.48 ± 0.01 ^ef^
15	99.79 ± 0.06 ^a^	0.41 ± 0.03 ^ef^	0.45 ± 0.03 ^bc^
Commercially available seasoning powder (Bonito soup stock)	89.70 ± 1.15 ^c^	-	
Maltodextrin	97.75 ± 0.31 ^cb^	-	

The values indicate the mean ± SD of triplicate determinations (*n* = 3). Different superscript lowercase letters in the same column represent significant differences between samples (*p* < 0.05).

**Table 4:** Sensory evaluation results of umami-rich seasoning powder and two commercially available products.

Sample		Appearance	Flavor					
		Color	Odor	Umami	Sweetness	Salty	Mouthfeel	Overall Preference
Umami-rich seasoning powder		7.67 ± 0.98 ^b^	7.33 ± 0.72 ^a^	7.53 ± 0.99 ^a^	7.40 ± 1.12 ^a^	7.60 ± 0.99 ^a^	7.47 ± 0.99 ^a^	7.53 ± 0.83 ^a^
Commercially available seasoning powder	1	7.07 ± 1.22 ^b^	7.13 ± 1.64 ^a^	7.00 ± 2.00 ^a^	6.87 ± 1.92 ^b^	6.73 ± 1.91 ^a^	6.40 ± 2.06 ^a^	6.73 ± 1.33 ^a^
	2	8.07 ± 0.80 ^a^	7.20 ± 0.68 ^a^	7.80 ± 0.86 ^a^	7.07 ± 1.53 ^c^	7.53 ± 0.99 ^a^	7.53 ± 1.06 ^a^	7.47 ± 0.99 ^a^

The values indicate the mean ± SD of triplicate determinations (*n* = 3). Different superscript lowercase letters in the same column represent significant differences between samples (*p* < 0.05).

## Data Availability

Data is contained within the article.

## Appendix A

**Table A1:** Experimental design for three variables—three levels of response surface analysis of umami-rich seasoning.

Independent Variables	Run	Coded	Uncoded
A: Maltodextrin content (g)	±1	1	500
	±1	0	400
	0	−1	300
	0		
B: Flow rate (rpm)	±1	1	6
	0	0	4
	±1	−1	2
	0		
C: Inlet temperature (°C)	0	1	80
	±1	0	70
	±1	−1	60
	0		
Number of runs	15		

**Table A2:** Analysis of the basic composition and physicochemical properties of tilapia’s soup stock, cooking liquid, and concentrated ones.

	Tilapia		
	Soup Stock	Cooking Liquid ^1^	Concentrated Liquid ^2^
Crude protein (%)	2.8 ± 0.01 ^a^	4.8 ± 0.01 ^b^	11.6 ± 0.01 ^c^
Crude fat (%)	0.2 ± 0.01 ^a^	N.D.	0.5 ± 0.05 ^b^
Moisture (%)	92.8 ± 0.02 ^a^	95.0 ± 0.02 ^b^	88.0 ± 1.00 ^c^
Ash (%)	0.1 ± 0.02 ^a^	0.4 ± 0.02 ^b^	1.1 ± 1.05 ^c^
Sugar content (°Brix)	3.33 ± 0.12 ^a^	6.93 ± 0.09 ^b^	14.27 ± 0.09 ^c^
Salinity (g/100 g)	3.00 ± 0.00 ^a^	5.73 ± 0.09 ^b^	12.47 ± 0.09 ^c^
pH value	6.42 ± 0.31 ^a^	6.06 ± 0.01 ^b^	5.99 ± 0.00 ^c^
*L**	0.90 ± 0.00 ^a^	4.31 ± 0.15 ^b^	0.57 ± 0.04 ^c^
*a**	0.47 ± 0.00 ^a^	0.62 ± 0.19 ^b^	0.31 ± 0.18 ^c^
*b**	1.56 ± 0.00 ^a^	4.19 ± 0.07 ^b^	0.98 ± 0.08 ^c^
ΔE	0.00 ± 0.00 ^a^	0.42 ± 0.01 ^b^	0.43 ± 0.00 ^c^

The values indicate the mean ± SD of triplicate determinations (*n* = 3). Lowercase letters in the same row represent significant differences between samples (*p* < 0.05). N.D., not detected. ^1^ The cooking liquid from the first steaming of tilapia (yield of about 16.67%) serves to produce fish-flavored soy sauce. ^2^ The concentrate refers to the sample obtained by vacuum rotary concentration from cooking liquid.

**Table A3:** The chromaticity of umami-rich seasoning powders with different carriers and the cost of carrier materials.

	*L**	*a**	*b**	Price (USD/25 Kg)
Maltodextrin	86.10 ± 0.73 ^b^	1.35 ± 0.16 ^a^	13.77 ± 0.46 ^b^	48.82 ^c^
Lactose	88.61 ± 0.06 ^a^	0.99 ± 0.11 ^b^	11.63 ± 0.07 ^c^	81.36 ^b^
Indigestible dextrin	84.68 ± 0.22 ^c^	1.21 ± 0.65 ^a^	15.46 ± 0.39 ^a^	136.69 ^a^

Values are given as ± SD from triplicate determinations (*n* = 3). Lowercase letters in the same column represent significant differences between samples (*p* < 0.05).

**Table A4:** Experimental responses were obtained under spray drying conditions with a three-factor, three-variable Box–Behnken experiment designed with statistical information on the defined umami-rich seasoning powders (yield and moisture content) with model fitting procedures.

Source	Sum of Squares	DF	Mean	*F*-Value	*p*-Value	
			Square			
Model	6.84	9	0.7599	5.67	0.0352	Significant
A: Maltodextrin content	0.726	1	0.726	5.42	0.0673	
B: Flow rate	1.58	1	1.58	11.77	0.0186	
C: Inlet temperature	1.8	1	1.8	13.48	0.0144	
AB	0.8836	1	0.8836	6.6	0.0501	
AC	0.5256	1	0.5256	3.93	0.1044	
BC	0.024	1	0.024	0.1795	0.6894	
A^2^	1.11	1	1.11	8.27	0.0348	
B^2^	0.2642	1	0.2642	1.97	0.219	
C^2^	0.0299	1	0.0299	0.2234	0.6563	
Residual	0.6693	5	0.1339			
Lack of fit	0.4047	3	0.1349	1.02	0.5298	Not significant
Pure error	0.2646	2	0.1323			
Cor Total	7.51	14				
Statistical data						
Std Dev.			0.3659			
Mean			4.77			
C.V.%			7.67			
R^2^			0.9109			
Adjusted R^2^			0.7504			
Predicted R^2^			0.0583			
Adeq precision			6.9169			

Y = −0.48A − 0.44B + 0.30C − 0.08AB + 0.36AC + 0.47BC + 0.55A^2^ + 0.27B^2^ + 0.09C^2^ + 4.29 = 5.33

**Table A5:** Experimental data for various responses of umami-rich seasoning powders with different combinations of maltodextrin content (A), flow rate (B), and inlet temperature (C) were used in the design for response surface methodology.

Run	A	B	C	Drying			
	Maltodextrin Content	Flow Rate	Inlet Temperature	Before	After	Yield	Moisture Content
Unit	g	rpm	°C	g	g	%	%
1	400	6	60	600	446	74.33	4.96
2	300	4	60	500	324	64.8	5.31
3	400	4	70	600	392	65.33	4.08
4	500	6	70	700	509	72.71	5.13
5	400	2	60	600	460	76.67	5.33
6	300	4	80	500	370	74	3.68
7	400	4	70	600	400	66.67	4.71
8	500	2	70	700	479	68.43	5.43
9	400	2	80	600	399	66.5	4.49
10	300	6	70	500	304.5	60.9	3.84
11	500	4	80	700	503	71.86	5.27
12	400	4	70	600	402	67	4.53
13	500	4	60	700	535	76.43	5.45
14	400	6	80	600	413	68.83	3.81
15	300	2	70	500	357.5	71.5	6.03
